# Increased Confusion and Memory Loss in Households, 2011 Behavioral Risk Factor Surveillance System

**DOI:** 10.5888/pcd12.140430

**Published:** 2015-03-05

**Authors:** Angela J. Deokar, Erin D. Bouldin, Valerie J. Edwards, Lynda A. Anderson

**Affiliations:** Author Affiliations: Erin D. Bouldin, VA Puget Sound Health Care System, Seattle, Washington; Valerie J. Edwards, Centers for Disease Control and Prevention, Atlanta, Georgia; Lynda A. Anderson, Centers for Disease Control and Prevention and Emory University, Atlanta, Georgia.

## Abstract

Using data from the 2011 Behavioral Risk Factor Surveillance System (BRFSS), we examined households in 13 states (N = 81,012) in which the respondent or another adult household member experienced increased confusion or memory loss (ICML) in the preceding 12 months. A total of 12.6% of households reported at least 1 adult who experienced ICML, and in 5.4% of households all adults experienced ICML. Based on these results, an estimated 4 million households in these 13 states have a member with ICML, potentially affecting more than 10 million people. This study can inform public health communication campaigns aimed at increasing awareness of the signs and symptoms of cognitive decline and augment community planning efforts so that the needs of households in which 1 or more adults has cognitive decline are considered.

## Objective

In 2011, the Behavioral Risk Factor Surveillance System (BRFSS) added questions about increased confusion or memory loss (ICML) experienced during the previous 12 months by adult(s) in the household. Thus, unique information about ICML at the household level is available. Our objective was to describe the number and characteristics of households that have members with ICML using available household-level variables (ie, income and number of adults and children in the household). Because memory problems can affect medication adherence and can cause safety concerns, we also described characteristics of households in which all adults (aged ≥18) experienced ICML.

## Methods

The BRFSS consists of annual state-based telephone surveys of randomly selected noninstitutionalized adults on health practices and risk behaviors. Details of the 2011 BRFSS and its response rates are available at http://www.cdc.gov/brfss/annual_data/annual_2011.htm. In BRFSS, 1 adult is randomly selected to represent each household. Our study focused on the household, defined as a unit consisting of all people occupying a residence intended for use as a separate living quarter.

Responses to 2 questions were used to determine the number of adults in the household who experienced ICML: 1) “During the past 12 months, have you experienced confusion or memory loss that is happening more often or is getting worse?,” and 2) “How many adults 18 years or older in your household [If Q1 = yes, other than yourself,] experienced confusion or memory loss that is happening more often or is getting worse during the past 12 months?”

Data were included from 13 states that had available household weights and included ICML questions on their 2011 surveys (Arkansas, Florida, Georgia, Hawaii, Illinois, Iowa, Louisiana, New Hampshire, North Carolina, South Carolina, Tennessee, West Virginia, and Wisconsin).

Cellular telephone and split-sample surveys were excluded because household weights were not available. Data on the reported number of people in a household with ICML were compared with data collected as part of the initial BRFSS selection process on the number of people in the household. Inconsistent reports were found in less than 0.3% of total households and were removed. Additionally, data on respondents with missing values for ICML were excluded.

Households (n = 81,012) were classified according to whether any person in the household experienced ICML and whether all persons in the household experienced ICML. Analyses were performed from January through May 2014 using Stata version 11 (StataCorp LP) and adjusted using household weights. We used the weighted estimates from the BRFSS along with data from the 2011 American Community Survey to estimate the number of households with members experiencing ICML and the number of people in those households.

## Results

A total of 12.6% (n = 10,537) of households reported at least 1 adult with ICML, and 5.4% of households reported that all of the adults had ICML (Table 1). In 6.9% (95% confidence interval [CI], 6.6%–7.1%; n = 5,712) of households, only the respondent had ICML; in 3.7% (95% CI, 3.5%–3.9%, n = 3,075), the respondent reported that at least 1 other adult had ICML; and in 2.0% (95% CI, 1.9%–2.2%, n = 1,750), the respondent and at least 1 other adult had ICML.

**Table 1:** Characteristics of Households in 13 States,^a^ by Number of Household Members With Increased Confusion or Memory Loss, 2011 BRFSS

Household Characteristic	All Households, Unweighted No.	Any Adult in Household Has ICML			All Adults in Household Have ICML		
		No. of Households, Unweighted	Weighted % (95% CI)	*P* Value^b^	Number of Households, Unweighted	Weighted % (95% CI)	*P* Value^b^
**All households**	81,012	10,537	12.6 (12.3–13.0)	NA	4,672	5.4 (5.2–5.7)	NA
**Annual income, $**							
<15,000	8,739	1,883	21.7 (20.3–23.1)	<.001	1,231	13.8 (12.8–15.0)	<.001
15,000–24,999	13,427	2,184	16.8 (15.8–17.8)		1,078	8.5 (7.7–9.3)	
25,000–49,999	19,324	2,526	13.3 (12.5–14.0)		981	4.9 (4.4–5.4)	
50,000–74,999	10,511	1,117	10.2 (9.4–11.2)		336	3.0 (2.5–3.5)	
≥75,000	17,173	1,356	7.2 (6.6–7.8)		366	2.0 (1.7–2.4)	
**No. of adults in household**							
1	29,086	3,231	11.2 (10.7–11.8)	<.001	3,231	11.2 (10.7–11.8)	<.001
2	41,797	5,384	12.0 (11.6–12.5)		1,364	2.9 (2.7–3.2)	
≥3	10,129	1,922	18.5 (17.3–19.7)		77	0.8 (0.6–1.1)	
**No. of children (aged <18 y) in household**							
0	61,805	8,556	14.0 (13.6–14.5)	<.001	4,074	6.6 (6.3–6.9)	<.001
≥1	19,055	1,967	9.8 (9.2–10.5)		589	3.1 (2.8–3.5)	

Abbreviations: BRFSS, Behavioral Risk Factor Surveillance System; CI, confidence interval; ICML, increased confusion or memory loss; NA, not applicable.

a Arkansas, Florida, Georgia, Hawaii, Illinois, Iowa, Louisiana, New Hampshire, North Carolina, South Carolina, Tennessee, West Virginia, and Wisconsin.

b *P* value for difference across categories within household type.

Among households with at least 2 adults, 13.4% (95% CI, 12.9%–13.8%, n = 7,306) had at least 1 adult with ICML. In 4.7% (95% CI, 4.5%–5.1%, n = 2,481) of households with at least 2 adults, only the respondent had ICML; in 5.5% (95% CI, 5.2%–5.8%, n = 3,075), the respondent reported that at least 1 other adult had ICML; and in 3.1% (95% CI, 2.9%–3.3%, n=1,750), the respondent and at least 1 other adult had ICML.

The percentage of households in which at least 1 adult had ICML increased with the number of adults in the household, ranging from 11.2% in households of 1 adult to 18.5% in households of 3 or more adults (Table 1). In households with children younger than 18, 9.8% had at least 1 adult with ICML. In 3.1% of households with children, all adults had ICML. The percentage of households with at least 1 adult with ICML was significantly lower among high-income households than among low-income households, ranging from 7.2% (95% CI, 6.6%–7.8%, n = 1,356) for annual household incomes of $75,000 or more to 21.7% (95% CI, 20.3%–23.1%, n = 1,883) for annual household incomes below $15,000. This trend was similar regardless of household size or composition (eg, number of children or number of women). In these 13 states, an estimated 3,979,666 households representing 10,353,268 people had at least 1 adult with ICML (Table 2).

**Table 2:** Estimates of the Number of Households With at Least 1 Adult Who Experienced Increased Confusion or Memory Loss During the Previous Year, by State, 13 US States, 2011

State	Weighted Estimate of Households With ICML^a^, %	No. of Households in State^b^	Estimated No. of Households With ICML	Average No. of People per Household (Among Households With ICML)^b^	Estimated No. of People Impacted by Household ICML
Arkansas	20.2	1,127,621	227,779	2.54	578,560
Florida	15.7	7,106,283	1,115,686	2.62	2,923,098
Georgia	13.8	3,494,542	482,247	2.74	1,321,356
Hawaii	13.5	448,563	60,556	2.97	179,851
Illinois	11.7	4,737,208	554,253	2.65	1,468,771
Iowa	9.7	1,216,765	118,026	2.44	287,984
Louisiana	9.1	1,702,030	154,885	2.61	404,249
New Hampshire	12.6	516,454	65,073	2.47	160,731
North Carolina	11.2	3,683,364	412,537	2.55	1,051,969
South Carolina	15.4	1,768,834	272,400	2.57	700,069
Tennessee	7.0	2,467,428	172,720	2.53	436,981
West Virginia	10.2	735,408	75,012	2.46	184,529
Wisconsin	11.8	2,275,352	268,492	2.44	655,119
**Total**	12.6	31,279,852	3,979,666	2.58	10,353,268

Abbreviation: ICML, increased confusion or memory loss.

a Estimate from Behavioral Risk Factor Surveillance System.

b Estimate from American Community Survey.

## Discussion

Our study is the first to report on ICML in households and provides a glimpse of the reach of ICML directly or indirectly into households. Other research on ICML in individuals and associations with functional difficulties and chronic conditions, using data from 21 states, is being reported in this issue by Anderson and colleagues (1). Because older adults with memory complaints have a greater risk than those without memory complaints for developing mild cognitive impairment (a potential precursor to Alzheimer’s disease) (2), households in which older adults have memory complaints could face health and financial consequences (3–5). Cognitive decline can lead to safety and health consequences and is particularly concerning for households in which the only adult has ICML.

Of all chronic conditions, brain-related conditions impose the greatest risk to the psychological well-being of other family members (6). Because children often provide care and support in families that experience chronic illnesses and disability (7), our findings suggest a need to consider their unique challenges when providing services and supports to households with children. Studies have demonstrated an association between individual-level (8,9) and neighborhood-level socioeconomic status (10,11) and cognitive decline. Here, we observed an association between household income and cognitive decline.

Our findings are subject to limitations. Data were collected from 13 states and cannot be construed as national averages. Data are self-reported and subject to bias, particularly when respondents report on “other” adult(s). The survey design is cross-sectional, so causality cannot be determined. The ICML measure is not clinically validated and may not correspond to specific diagnoses. Finally, using household weights restricted analyses to household-level variables. Additional questions for households would enhance future research.

These findings highlight the magnitude of the problem of cognitive decline and can help inform public health programs and policies. For example, increasing awareness about recognition of signs and symptoms of cognitive decline in self or others can allow household members to seek medical advice and plan for future needs.
